# Systematic Histological Scoring Reveals More Prominent Interstitial Inflammation in Myeloperoxidase-ANCA Compared to Proteinase 3-ANCA Glomerulonephritis

**DOI:** 10.3390/jcm10061231

**Published:** 2021-03-16

**Authors:** Samy Hakroush, Ingmar Alexander Kluge, Philipp Ströbel, Peter Korsten, Désirée Tampe, Björn Tampe

**Affiliations:** 1 Institute of Pathology, University Medical Center Göttingen, 37075 Göttingen, Germany; samy.hakroush@med.uni-goettingen.de (S.H.); ingmar.kluge@med.uni-goettingen.de (I.A.K.); philipp.stroebel@med.uni-goettingen.de (P.S.); 2 Department of Nephrology and Rheumatology, University Medical Center Göttingen, 37075 Göttingen, Germany; peter.korsten@med.uni-goettingen.de (P.K.); desiree.tampe@med.uni-goettingen.de (D.T.)

**Keywords:** ANCA-associated vasculitis, small vessel vasculitis, ANCA glomerulonephritis, ANCA subtype, MPO, PR3, tubulointerstitial lesions, inflammation, arteritis

## Abstract

Background: Antineutrophil cytoplasmic antibody (ANCA)-associated vasculitis (AAV) is a systemic vasculitis, most frequently presenting as microscopic polyangiitis (MPA) or granulomatosis with polyangiitis (GPA). Kidney involvement is a common and severe complication of ANCA AAV which is observed in a considerable subset of patients, mainly affecting glomeruli. However, tubulointerstitial lesions have also been described in ANCA glomerulonephritis (GN). Therefore, we aim to describe active and chronic tubulointerstitial lesions in ANCA GN subtypes by systematic scoring analogous to the Banff scoring system while also utilizing clinical and laboratory findings. Methods: A total of 49 kidney biopsies with ANCA GN were retrospectively included in a single-center cohort study between 2015–2020. Results: We report that MPO-ANCA GN is associated with more severe deterioration of kidney function independent of systemic markers of AAV disease activity, and is also associated with increased proteinuria in MPO-ANCA GN and a decreased fraction of normal glomeruli. Finally, MPO-ANCA GN showed distinct, active, and chronic tubulointerstitial lesions. Conclusion: New insights into the pathophysiology of both entities, as well as differences in the clinical presentation of MPO- versus PR3-ANCA GN, could potentially pave the way for more precise treatment regimens. Therefore, it is important to understand the differences in histopathological presentation, especially in yet underestimated active tubulointerstitial lesions of ANCA GN subtypes. This research could further improve our understanding of distinct pathophysiological mechanisms.

## 1. Introduction

The presence of antineutrophil cytoplasmic antibodies (ANCAs) was described more than 50 years ago [1]. Subsequently, ANCAs were discovered in the context of associated vasculitis (AAV), a small vessel vasculitis affecting multiple organ systems, including the kidneys. ANCAs predominantly manifest as crescentic ANCA glomerulonephritis (GN) [2]. The discovery of ANCAs in granulomatosis with polyangiitis (GPA) marked a breakthrough in diagnostics, since GPA had long been known without a serologic marker [3,4]. Subsequently, ANCAs were found in other forms of small vessel vasculitis, microscopic polyangiitis (MPA), and eosinophilic granulomatosis with polyangiitis (EGPA) [5,6]. Three patterns of ANCAs can be detected by immunofluorescence techniques: cytoplasmic (c-ANCA), perinuclear (p-ANCA), and atypical (also called x-ANCA) [7]. While c-ANCA or p-ANCA are detected by immunofluorescence, a separate test is needed to detect proteinase 3 (PR3)-ANCA or myeloperoxidase (MPO)-ANCA, and is mandatory for the diagnosis of ANCA-associated vasculitis (AAV) [7]. Generally, renal manifestations in AAV are estimated at 80% among all cases mainly affecting glomeruli, and the overall prevalence does not seem to differ substantially between MPO-ANCA and PR3-ANCA AAV [8].

Current prognostic scoring systems of ANCA GN classify kidney biopsies based on the percentage of affected glomeruli into four categories: focal, crescentic, sclerotic, and mixed [9]. This classification has prognostic value with the best renal survival in the focal group, followed by the mixed and crescentic groups, and the worst renal survival in the sclerotic group [9,10,11]. With regard to ANCA GN subtypes, more fibrotic lesions have been observed previously in MPO-ANCA GN [12,13]. Tubulointerstitial lesions have also been described in ANCA GN. In this context, the ANCA renal risk score (ARRS) includes interstitial fibrosis, tubular atrophy, and the loss of estimated glomerular filtration rate (eGFR) for the assessment of renal outcomes [14]. Furthermore, interstitial inflammation is more pronounced in MPO-ANCA than in PR3-ANCA GN, further supporting the hypothesis that interstitial lesions differ between ANCA GN subtypes [15]. To gain further insight into distinct tubulointerstitial lesions in MPO-ANCA versus PR3-ANCA GN, we here aim to describe active and chronic tubulointerstitial lesions in ANCA GN subtypes by a systematic histological scoring analogous to the Banff scoring system. We also compare clinical and laboratory findings in a cohort of patients with ANCA GN [16].

## 2. Materials and Methods

### 2.1. Study Population

A total of 49 kidney biopsies with ANCA GN were retrospectively included in the sample. They were acquired from the University Medical Center Göttingen between 2015–2020. The patient cohort has, in part, been previously described [17]. Data regarding age, sex, diagnosis (MPA or GPA), and laboratory results were acquired from medical records. The glomerular filtration rate (GFR) was estimated using the Chronic Kidney Disease Epidemiology Collaboration (CKD-EPI) equation [18].

### 2.2. Definitions

At admission, the Birmingham Vasculitis Activity Score (BVAS) version 3 was calculated as described previously [19]. The BVAS was assessed on a scale of 0 to 63, with a score of 0 indicating the absence of any disease activity and higher scores indicating active disease. Since we aimed to explore the association between MPO-/PR3-ANCA and histopathological findings, we used the predominant ANCA subtype for group separation in our analyses.

### 2.3. Renal Histopathology

Renal pathologists (S.H. and P.S.) evaluated all biopsies and were blinded to all clinical data and related analyses. Within a kidney biopsy specimen, each glomerulus was scored separately for the presence of necrosis, crescents, and global sclerosis. Consequently, the percentage of glomeruli with any of these features was calculated as a fraction of the total number of glomeruli in each kidney biopsy. Based on these scorings, histopathological subgrouping according to Berden et al. (focal, crescentic, mixed, or sclerotic class) and ARRS according to Brix et al. (low, medium, or high risk) were performed [9,14]. Kidney biopsies were also evaluated analogous to the Banff scoring system for allograft pathology [16]. In brief, Banff score lesions included interstitial inflammation (*i*), tubulitis (*t*), arteritis (*v*), glomerulitis (*g*), interstitial fibrosis (*ci*), tubular atrophy (*ct*), arteriolar hyalinosis (*ah*), peritubular capillaritis (*ptc*), total inflammation (*ti*), inflammation in areas of IFTA (*i-IFTA*), and tubulitis in areas of IFTA (*t-IFTA*). The Banff scoring system had three grades: none (0), mild (1), moderate (2), and severe (3). The cut-off points for *i* were <10%, 10–25%, 26–50%, and >50%, respectively. Cut-off points for *t* were 0, 1–4, 5–10, and >10 mononuclear cells/tubular cross-section. Cut-off points for *v* were no arteritis, mild to moderate intimal arteritis in at least one arterial cross-section, severe intimal arteritis with at least 25% luminal area lost in at least one arterial cross-section, and transmural arteritis and/or arterial fibrinoid change and medial smooth muscle necrosis with lymphocytic infiltrate in vessel, respectively. Cut-off points for *g* were no glomerulitis, segmental or global glomerulitis in less than 25% of glomeruli, segmental or global glomerulitis in 25 to 75% of glomeruli, and segmental or global glomerulitis in more than 75% of glomeruli. Cut-off points for *ci* were interstitial fibrosis in up to 5%, 6–25%, 26–50%, and >50% of the cortical area. Cut-off points for *ct* were no tubular atrophy, tubular atrophy involving up to 25%, 26–50%, and >50% of the area of cortical tubules. Cut-off points for *ah* were no PAS-positive hyaline arteriolar thickening, mild to moderate PAS-positive hyaline thickening in at least one arteriole, in more than one arteriole, and in many arterioles. Cut-off points for *ptc* were a maximum number of leukocytes <3, at least one leukocyte cell in ≥10% of cortical PTCs with 3–4 leukocytes in most severely involved PTC, at least one leukocyte in ≥10% of cortical PTC with 5–10 leukocytes in most severely involved PTC, and at least one leukocyte in ≥10% of cortical PTC with >10 leukocytes in most severely involved PTC. Cut-off points for *ti* were <10%, 10-25%, 26-50%, and >50% of total cortical parenchyma inflamed. Cut-off points for *i-IFTA* and *t-IFTA* were no inflammation or less than 10%, 10–25%, 26–50%, and >50% of scarred cortical parenchyma.

### 2.4. Statistical Methods

Variables were tested for normal distribution using the Shapiro-Wilk test. Non-normally distributed continuous variables are expressed as the median and interquartile range (IQR), while categorical variables are presented as frequency and percentage. Statistical comparisons were not formally powered or prespecified. For group comparisons, the Mann-Whitney *U*-test was used to determine differences in medians. Nonparametric between-group-comparisons were performed with Pearson’s Chi-square test. Data analyses were performed with GraphPad Prism (version 8.4.3 for MacOS, GraphPad Software, San Diego, CA, USA).

## 3. Results

### 3.1. Study Population

**Figure 1:**
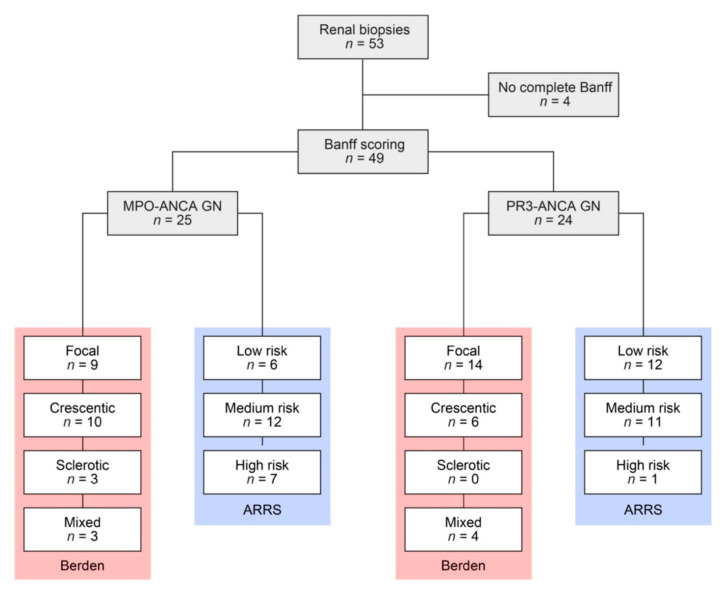
Total patient cohort of ANCA GN. STROBE flow chart of patient disposition included in this study. ANCA, antineutrophil cytoplasmic antibodies; ARRS, ANCA renal risk score; MPO, myeloperoxidase; PR3, proteinase 3; STROBE, Strengthening the Reporting of Observational Studies in Epidemiology.

A total number of 49 kidney biopsies with ANCA GN were retrospectively included (Figure 1 and Table 1).

### 3.2. MPO-ANCA GN Shows More Severe Deterioration of Kidney Function Independent of Systemic Markers of AAV Disease Activity

**Figure 2:**
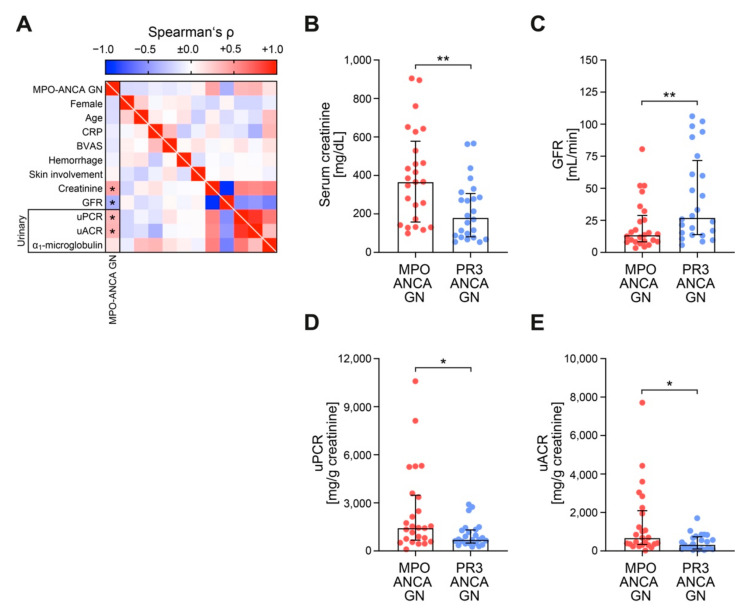
MPO-ANCA GN associates with more severe deterioration of kidney function independent of systemic markers of AAV disease activity. (**A**) Association between MPO-ANCA GN, clinical and laboratory findings are shown by heatmap reflecting mean values of Spearman’s ρ, asterisks indicate *p* < 0.05. (**B**–**E**) The scatter dot plots represent medians and IQR with individual data points summarizing association between MPO-ANCA GN, serum creatinine, GFR, uPCR and uACR. The Mann-Whitney *U*-test was used to determine differences in medians and asterisks indicate * *p* < 0.05, ** *p* < 0.01. ANCA, antineutrophil cytoplasmic antibodies; BVAS, Birmingham Vasculitis Activity Score; CRP, C-reactive protein; GFR, glomerular filtration rate (CKD-EPI); GN, glomerulonephritis; IgG, immunoglobulin G; IQR, interquartile range; MPO, myeloperoxidase; PR3, proteinase 3; uACR, urinary albumin-to-creatinine ratio; uPCR, urinary protein-to-creatinine ratio.

We first compared ANCA GN subtypes with regard to clinical and laboratory findings at the time of biopsy (Table 2). Whereas ANCA GN subtypes did not differ in age, gender, or systemic markers of AAV disease activity (C-reactive protein/CRP, Birmingham Vasculitis Activity Score/BVAS), MPO-ANCA GN was associated with more severe deterioration of kidney function as estimated by serum creatinine and loss of eGFR (Figure 2A–C). Among urinary findings, MPO-ANCA GN was associated with an increased total urinary protein-to-creatinine ratio (uPCR), including albumin-to-creatinine ratio (uACR) and urinary IgG and α2-macroglobulin (Figure 2A,D,E), implicating nonselective glomerular proteinuria. In contrast, tubular proteinuria, reflected by α1-microglobulin, did not differ between ANCA GN subtypes (Figure 2A).

### 3.3. Histology of MPO-ANCA GN Reveals Decreased Fraction of Normal Glomeruli

**Figure 3:**
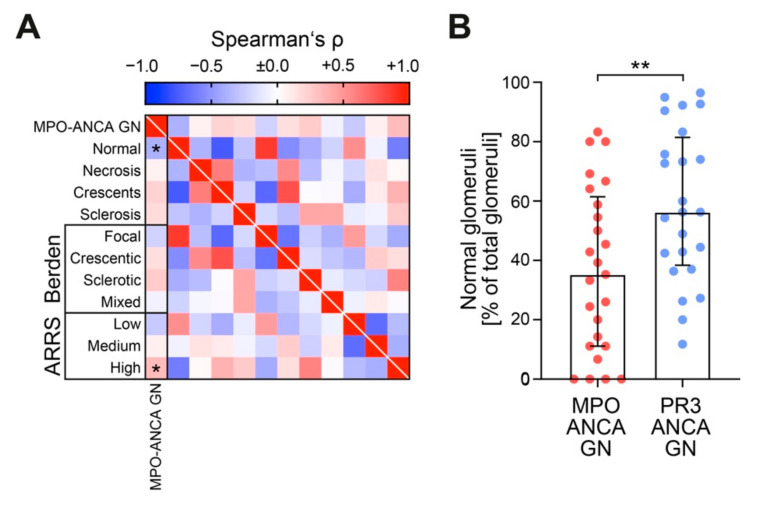
MPO-ANCA GN associates with decreased fraction of normal glomeruli and glomerular lesions are equally observed in ANCA GN subtypes. (**A**) Association between MPO-ANCA GN and glomerular findings are shown by heatmap reflecting mean values of Spearman’s ρ, asterisks (*) indicate *p* < 0.05. (**B**) The scatter dot plots represent medians and IQR with individual data points summarizing association between MPO-ANCA GN and fraction of normal glomeruli. The Mann-Whitney *U*-test was used to determine differences in medians and asterisks indicate ** *p* < 0.01. ANCA, antineutrophil cytoplasmic antibodies; GN, glomerulonephritis; IQR, interquartile range; MPO, myeloperoxidase; PR3, proteinase 3.

Since we observed a more severe decline of kidney function and accelerated proteinuria in MPO-ANCA GN, we then analyzed glomerular lesions in both ANCA GN subtypes (Table 3). Normal glomeruli decreased; however, this decrease could not be attributed to distinct glomerular lesions (Figure 3A,B). Therefore, this finding confirmed that glomerular lesions are equally observed in both ANCA GN subtypes, while decreased normal glomeruli are more frequently encountered in MPO-ANCA GN [12,20,21,22,23]. Using the established ANCA scoring systems, MPO-ANCA GN was classified more frequently as ARRS high risk including loss of eGFR (Figure 3A), consistent with our aforementioned findings, i.e., that MPO-ANCA GN showed more severe deterioration of kidney function.

### 3.4. Banff Scoring Reveals Prominent Interstitial Vasculitis and Inflammation in MPO-ANCA GN

**Figure 4:**
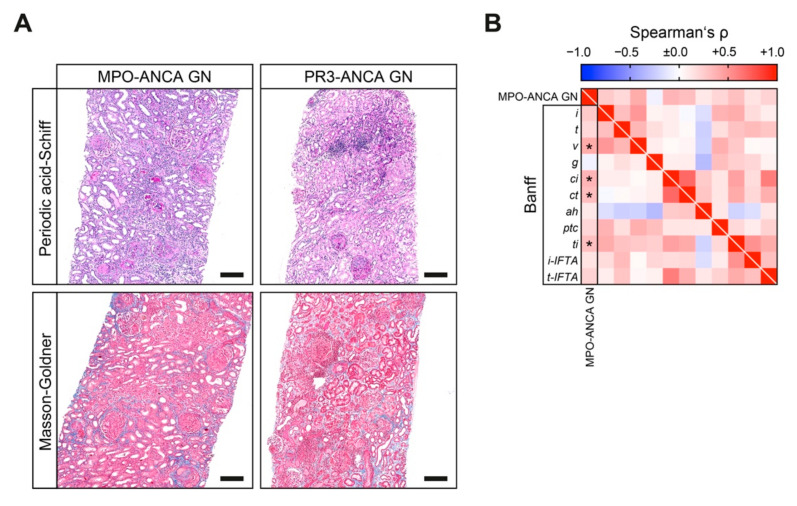
MPO-ANCA GN associates with interstitial vasculitis and inflammation, interstitial fibrosis and tubular atrophy. (**A**) Representative periodic acid-Schiff and Masson-Goldner-stained kidney sections of MPO-ANCA and PR3-ANCA GN are shown (scale bars: 200 μm). (**B**) Association between MPO-ANCA compared to PR3-ANCA GN with regard of tubulointerstitial findings analogous to the Banff scoring system are shown by heatmap reflecting mean values of Spearman’s ρ, asterisks (*) indicate *p* < 0.05. *ah*, arteriolar hyalinosis; ANCA, antineutrophil cytoplasmic antibodies; *ci*, interstitial fibrosis; *ct*, tubular atrophy; *g*, glomerulitis; GN, glomerulonephritis; *i*, interstitial inflammation; *i-IFTA*, inflammation in IFTA; IQR, interquartile range; MPO, myeloperoxidase; PR3, proteinase 3; *t*, tubulitis; *ptc*, peritubular capillaritis; *ti*, total inflammation; *t-IFTA*, tubulitis in IFTA; *v*, arteritis.

We next analyzed tubulointerstitial lesions analogous to the Banff scoring system in both ANCA GN subtypes (Table 4). We found interstitial fibrosis (*ci*) and tubular atrophy (*ct*) increased in MPO-ANCA as compared to PR3-ANCA GN (Figure 4A,B). Interestingly, MPO-ANCA GN exhibited more active tubulointerstitial lesions, as reflected by higher Banff lesions scores for interstitial arteritis (*v*) and total inflammation (*ti*, Figure 4A,B).

## 4. Discussion

To our knowledge, this is the largest study to date on AAV that reports the systematic histological scoring of tubulointerstitial lesions in MPO-ANCA and PR3-ANCA GN compared to clinical and laboratory findings. Differences between AAV subtypes have been previously described: PR3-AAV is characterized by predominant involvement of the upper respiratory tract, whereas MPO-AAV less frequently affects the lower respiratory tract and the kidneys [24,25]. Both entities present with a similar renal histomorphology, while a reduced number of normal glomeruli and more interstitial fibrosis reflecting chronic damage have been observed in MPO-ANCA GN. This finding potentially explains the reduced rate of renal recovery in MPA [26,27].

Here, we report that MPO-ANCA GN correlates with more severe deterioration of kidney function independent of systemic markers of AAV disease activity. Furthermore, higher levels of proteinuria were observed in MPO-ANCA GN; this was mainly attributed to nonselective glomerular proteinuria, which is in line with previous reports [28]. By comparing glomerular findings, normal glomeruli decreased, but were not attributed to distinct glomerular lesions. Finally, MPO-ANCA GN showed significantly more interstitial vasculitis and total inflammation, as well as interstitial fibrosis and tubular atrophy. These observations are of interest and require further interpretation, as severe kidney injury at disease onset (as we observed in MPO-ANCA GN) has been associated with poor long-term renal outcome in ANCA GN [29]. In addition, more severe proteinuria (again, as we observed in MPO-ANCA GN) has also been connected to poorer outcomes [13,28,30,31]. Finally, increased chronic tubulointerstitial lesions reflected by interstitial fibrosis and tubular atrophy (as we observed in MPO-ANCA GN) have previously been shown to correlate with worse renal outcome, and therefore, have been implemented into established scoring systems in ANCA GN [12,13,14]. While these observations suggest that MPO-ANCA GN may also have worse renal outcomes, data on this matter remain inconclusive [20,32,33,34]. Interestingly, histopathological examination of MPO-ANCA GN revealed more active tubulointerstitial lesions, as reflected by interstitial vasculitis and total inflammation. While the aforementioned chronic damage potentially explains the observed reduction of renal recovery in MPA, interstitial vasculitis and inflammation are, in principle, responsive to aggressive immunosuppressive regimens [26,27]. Tubulointerstitial inflammation has previously been associated with active glomerular lesions [15]. Interstitial inflammation is more pronounced in MPO-ANCA than in PR3-ANCA G, is associated with tubular lesions, and is localized to severely inflamed glomeruli or vessels [15]. Less common is an interstitial granulomatous inflammation not localized to glomeruli and characterized by a central zone of necrosis surrounded by an infiltrate of mononuclear leukocytes, neutrophils, and scattered multinucleated giant cells. Therefore, understanding differences in histopathological presentation, especially in yet underestimated active tubulointerstitial lesions of ANCA GN subtypes, could be the first step toward improving our understanding of distinct pathophysiological mechanisms and specific treatment strategies.

The main limitations of our study are its retrospective design and lack of data on long-term renal survival rates. Nevertheless, our finding that tubulointerstitial lesions may differ in MPO-ANCA compared to PR3-ANCA GN is of interest and requires further investigation as well as independent validation. Specifically, these findings are of great clinical utility and may guide physicians to identify which patients may benefit the most from aggressive and potentially tailored immunosuppressive therapy. These findings are of potential interest for therapeutic decision-making in ANCA GN subtypes presenting with different clinical and histopathological features.

## 5. Conclusions

In summary, we report that MPO-ANCA GN is associated with a more severe deterioration of kidney function independent of systemic markers of AAV disease activity, with increased proteinuria in MPO-ANCA GN, and with a decreased fraction of normal glomeruli. Additionally, MPO-ANCA GN showed distinct active and chronic tubulointerstitial lesions. Therefore, the characterization of differences in ANCA GN subtypes could further improve our understanding of distinct pathophysiological mechanisms and specific treatment strategies.

## Figures and Tables

**Table 1:** Total patient cohort of ANCA GN.

Parameter	Value
No. of kidney biopsies	49
Clinical data	
Female sex—no. (%)	22 (44.9)
Age (IQR)—years	66 (55–74.5)
Disease activity	
CRP (IQR)—mg/L	63.6 (23.4–109)
BVAS (IQR)—points	18 (15–21)
Pulmonary hemorrhage—no. (%)	7 (14.3)
Skin involvement—no. (%)	7 (14.3)
Kidney injury	
Serum creatinine (IQR)—μmol/L	272 (123–437)
GFR (IQR)—mL/min/1.73 m^2^	17.6 (9.7–47.9)
Urinary findings	
uPCR (IQR)—mg/g creatinine	977 (573–1939)
uACR (IQR)—mg/g creatinine	458 (202–938)
α_1_-microglobulin (IQR)—mg/g creatinine	69.6 (34.9–174)
Glomerular lesions	
Median total glomeruli (IQR)—no.	17 (11–28)
Median normal glomeruli (IQR)—%	45.5 (25.2–73)
Median glomerular necrosis (IQR)—%	15.2 (0–44.7)
Median glomerular crescents (IQR)—%	33.3 (10–55.1)
Median glomerular sclerosis (IQR)—%	5.1 (0–26.5)
Berden score	
Focal class—no. (%)	23 (46.9)
Crescentic class—no. (%)	16 (32.7)
Sclerotic class—no. (%)	3 (6.1)
Mixed class—no. (%)	7 (14.3)
ARRS	
Low risk—no. (%)	18 (36.7)
Medium risk—no. (%)	23 (46.9)
High risk—no. (%)	8 (16.3)

ANCA, antineutrophil cytoplasmic antibodies; BVAS, Birmingham Vasculitis Activity Score; CRP, C-reactive protein; GFR, glomerular filtration rate (CKD-EPI); IQR, interquartile range; MPO, myeloperoxidase; No., number; PR3, proteinase 3; uACR, urinary albumin-to-creatinine ratio; uPCR, urinary protein-to-creatinine ratio. ANCA, antineutrophil cytoplasmic antibodies; ARRS, ANCA renal risk score; GN, glomerulonephritis; MPO, myeloperoxidase; No., number; PR3, proteinase 3.

**Table 2:** ANCA GN subtypes with regard of clinical and laboratory findings.

	MPO-ANCA GN	PR3-ANCA GN	*p* Value
No. of kidney biopsies	25	24	0.2012
Clinical data			
Female sex—no. (%)	9 (36)	13 (54)	0.2012
Age (IQR)—years	61 (52–70)	69.5 (55–76)	0.2222
Disease activity			
CRP (IQR)—mg/L	57.4 (24–102)	67.6 (21.9–118)	0.5354
BVAS (IQR)—points	18 (14–21)	19 (16–20.8)	0.3929
Pulmonary hemorrhage—no. (%)	3 (12)	4 (16.7)	0.4607
Skin involvement—no. (%)	4 (16)	3 (12.5)	0.7263
Kidney injury			
Serum creatinine (IQR)—μmol/L	367 (158–578)	181 (80.9–306)	**0.0027**
GFR (IQR)—mL/min/1.73 m^2^	13.7 (8.3–28.8)	27.2 (14–71.6)	**0.0076**
Urinary findings			
uPCR (IQR)—mg/g creatinine	1446 (671–3476)	719 (490–1319)	**0.0199**
uACR (IQR)—mg/g creatinine	692 (337–2097)	338 (103–739)	**0.0119**
α_1_−microglobulin (IQR)—mg/g creatinine	101 (34.9–205)	62.7 (36.7–122)	0.3479

For group comparisons, the Mann-Whitney *U*-test was used to determine differences between medians. Nonparametric between-group-comparisons were performed with Pearson’s Chi-square test. Median values are shown, bold indicates statistically significant values at group level. ANCA, antineutrophil cytoplasmic antibodies; BVAS, Birmingham Vasculitis Activity Score; CRP, C-reactive protein; GFR, glomerular filtration rate (CKD-EPI); IQR, interquartile range; MPO, myeloperoxidase; No., number; PR3, proteinase 3; uACR, urinary albumin-to-creatinine ratio; uPCR, urinary protein-to-creatinine ratio.

**Table 3:** ANCA GN subtypes with regard to glomerular findings.

	MPO-ANCA GN	PR3-ANCA GN	*p* Value
No. of kidney biopsies	25	24	
Glomerular lesions			
Median normal glomeruli (IQR)—%	35.3 (11.1–61.5)	56.3 (38.4–81.4)	**0.0071**
Median glomerular necrosis (IQR)—%	16.7 (0–48.3)	14.2 (0.9–43.5)	0.6704
Median glomerular crescents (IQR)—%	37.5 (14.8–69.1)	27.9 (8.2–49.7)	0.1355
Median glomerular sclerosis (IQR)—%	10 (0–38.2)	4.4 (0–17.2)	0.2401
Berden score			
Focal class—no. (%)	9 (36)	14 (58.3)	
Crescentic class—no. (%)	10 (40)	6 (25)	
Sclerotic class—no. (%)	3 (12)	0 (0)	
Mixed class—no. (%)	3 (12)	4 (16.7)	0.1569
ARRS			
Low risk—no. (%)	6 (24)	12 (50)	
Medium risk—no. (%)	12 (48)	11 (45.8)	
High risk—no. (%)	7 (28)	1 (4.2)	**0.0383**

For group comparisons, the Mann-Whitney *U*-test was used to determine differences between medians. Nonparametric between-group-comparisons were performed with Pearson’s Chi-square test. Median values are shown, bold indicates statistically significant values at group level. ANCA, antineutrophil cytoplasmic antibodies; ARRS, ANCA renal risk score; GN, glomerulonephritis; MPO, myeloperoxidase; No., number; PR3, proteinase 3.

**Table 4:** Tubulointerstitial findings associated with MPO-ANCA compared to PR3-ANCA GN.

Banff Lesion Score	Spearman’s ρ	*p* Value
Interstitial inflammation: *i*	0.254	0.0783
Tubulitis: *t*	0.182	0.2104
Arteritis: *v*	0.401	**0.0085**
Glomerulitis: *g*	−0.065	0.6559
Interstitial fibrosis: *ci*	0.351	**0.0135**
Tubular atrophy: *ct*	0.316	**0.0272**
Arteriolar hyalinosis: *ah*	0.109	0.4675
Peritubular capillaritis: *ptc*	0.195	0.1784
Total inflammation: *ti*	0.305	**0.0330**
Inflammation in areas of IFTA: *i-IFTA*	0.130	0.3742
Tubulitis in areas of IFTA: *t-IFTA*	0.209	0.1496

Bold indicates statistically significant values. ANCA, antineutrophil cytoplasmic antibodies; GN, glomerulonephritis; IFTA, interstitial fibrosis and tubular atrophy; MPO, myeloperoxidase; No., number.

## Data Availability

Deidentified data are available on reasonable request from the corresponding author.
